# Strategies to Improve Activity Recognition Based on Skeletal Tracking: Applying Restrictions Regarding Body Parts and Similarity Boundaries †

**DOI:** 10.3390/s18051665

**Published:** 2018-05-22

**Authors:** Carlos Gutiérrez-López-Franca, Ramón Hervás, Esperanza Johnson

**Affiliations:** MAmI Research Lab, University of Castilla-La Mancha, Paseo de la Universidad 4, 13071 Ciudad Real, Spain; ramon.hlucas@uclm.es (R.H.); mesperanza.johnson@alu.uclm.es (E.J.)

**Keywords:** activity recognition, Kinect, ubiquitous computing, ambient intelligence, extended body-angles algorithm

## Abstract

This paper aims to improve activity recognition systems based on skeletal tracking through the study of two different strategies (and its combination): (a) specialized body parts analysis and (b) stricter restrictions for the most easily detectable activities. The study was performed using the *Extended Body-Angles Algorithm*, which is able to analyze activities using only a single key sample. This system allows to select, for each considered activity, which are its *relevant joints*, which makes it possible to monitor the body of the user selecting only a subset of the same. But this feature of the system has both advantages and disadvantages. As a consequence, in the past we had some difficulties with the recognition of activities that only have a *small* subset of the joints of the body as relevant. The goal of this work, therefore, is to analyze the effect produced by the application of several strategies on the results of an activity recognition system based on *skeletal tracking joint oriented devices*. Strategies that we applied with the purpose of improve the recognition rates of the activities with a small subset of relevant joints. Through the results of this work, we aim to give the scientific community some first indications about which considered strategy is better.

## 1. Introduction

The analysis and recognition of human activities is a very popular area of work and research. Due to this fact, there are an innumerable amount of proposals in this research area with several types of focus and devices: machine learning methods, video analysis, mobile devices, wearable sensors and many more. Even though there are a variety of approaches when attempting to overcome the problem of activity recognition, there are some similarities as well. For example, most of the existing works are usually based on machine learning techniques.

On the other hand, it is not as typical to focus on analyzing certain aspects or strategies applicable to different systems, with the goal of improving recognition rates. As an example, there is the analysis of the effect that different body parts have on the results of activity recognition systems [1]. Though some of the existing works focus their analysis on *specific body parts* [2], it is not common to evaluate the existing differences between using some parts of the body or not [3,4]. These precedents demonstrate that it is interesting to center the analysis *only on specific parts of the body*.

The *Extended Body-Angles Algorithm* (*E-BA-A*) [5] is an activity recognition system that allows one to compare the similarity between two *postures* or *movements* performed by human bodies. This system, following the taxonomy of David and Marcus [6], is considered a case-based commonsense reasoning approach based on mathematical analogies. This kind of algorithm has only very limited interaction with machine learning approaches, that typically work over large data corpora. In fact, the main feature that differentiates this activity recognition system from others is that the ***E****-BA-A* doesn’t need to use a dataset with multiple instances of each supported activity. It is capable of performing the analysis with a *single instance* thanks to the use of the *angles* that are formed by each pair of joints as its unit of information. Another of its main features is the possibility to personalize which body parts are relevant in each activity. This way, we can optimize the computational cost by frame of the algorithm, omitting the processing of the irrelevant body parts. Taking into account the complexity of the *E-BA-A*, its functioning is explained more in depth in a previous publication [5]. In it, we also cover the aspects related to the comparison between the *E-BA-A* and the most common used recognition methods (based on machine learning techniques).

The goal of this work is to analyze the effect that is produced by the application of several strategies on the results of an activity recognition system. Specifically, on an activity recognition system based on the use of *skeletal tracking joint oriented devices*. As its name suggests, these devices perform the skeletal analysis tracking the 3D-position of the joints of the body. Therefore, this kind of device outputs the position in the environment of each one of the supported joints. We have used *Microsoft Kinect* as our sensor device and the *E-BA-A* as the activity recognition system in which we have applied the considered strategies of improvement. The extrapolation to other systems will be addressed in the discussion (Section 7).

In order to achieve this goal, in first place, we will expose which were the circumstances that led us to need to apply an improvement strategy in this kind of system. Later we will detail each one of the strategies applied, as well as the obtained results in each evaluation. The improvement strategies we have considered in this study are:

(1) Study how the use of different body parts affects the results in order to select as relevant only those joints that contribute to the improvement of the recognition.
(2) Apply greater restrictions (establish a minimum similarity limit that has to be surpassed) to those activities that are detected most easily through skeletal tracking joint oriented devices.
(3) Combine the use of the two previous strategies.

The article has following structure: Section 2 will explore in depth the contemplated state of the art; Section 3 will expose the difficulties that motivated the search of strategies to improve recognition rates; Section 4, Section 5 and Section 6 will describe each one of the strategies applied and the tests that have been performed (usually following the structure: *Description of the Strategy*, *Description of the Strategy Evaluation*, *Evaluation Results* and *Analysis of the Results*); the discussion will be presented in Section 7; and finally Section 8 will cover the final conclusions extracted from this study and the future work. In addition, the article has the following appendixes: Appendix A considers activities during the performance of the evaluations; Appendix B gives the body information of the volunteers; Appendix C describes the hardware used during the evaluations; and Appendix D covers particularities of the evaluations.

## 2. State of the Art

There are a wide range of proposals centered on the area of *activity recognition*, as it is a topic of study that generates a lot of interest. There are proposals to evaluate the performance of a dancer [7], to allow physical rehabilitation [8], activity recognition for the physical rehabilitation through mobile devices with accelerometer [9], detection of elderly frailty with accelerometry [10], detection of stereotypical behavior in autistic children [11], a review of human activity recognition methods and different examples of each method [12], an analysis of the current outlook of research in the field of activity recognition [13] and a system that monitors the activities that occur in an isolation room of a psychiatric hospital [14].

There are also proposals that support themselves on *objects from the environment* (taking context into account) to do the activity analysis. Some of those examples are monitoring the activities and state of a dog through sensors embedded in their collar [15], a system of activity recognition of daily living that takes context into account (the objects the user interacts with) [16] and lastly the detection of activities done in a house with simple ubiquitous sensors [17].

Other kinds of proposals center around the use of *alternative recognition strategies*, like the recognition of activities in which the observer in 1st person takes part [18], a study of activity recognition when information from skeleton tracking is not available [19], or gesture recognition [20] and hand gesture [21].

Some of the studied works analyze activities focusing only on a *specific body part*. Some examples are a system to guide people with dementia in the performance of activities of daily living [22], a system based on context centered around culinary activities and objects [23] and a method capable of recognize activities from a video camera in an egocentrical point of view analyzing the hands of the user and the objects [2]. But, in this group of works, those that approach the possible differences in recognition are mostly related with *variations in the disposition of the accelerometers* on the human body. There are studies that analyze the impact that these variations of the accelerometers from their initial position in which they were put have [24,25], studies about the *optimal placement position of accelerometers* and how to improve recognition combining the data from several of them [3] and a method capable of *obtaining the position of the body* in which an accelerometer has been placed based only on the signal of the device [26] (as knowing where the sensor is located is very important information about context).

Lastly, most similar related work to our proposal corresponds to two proposals opposite to each other. The first of them proposes to analyze human activities distinguishing among the different body parts of the person and applying a relational representation by means of graphs [4]. This way they attempt to test if this representation obtains better results than considering the whole body as a single object. The second proposal aims for the opposite [27] and maintains that it is possible to recognize repetitive activities without discerning between the different parts that compose the human bodies. For this, they present a method capable of performing this analysis through sequences of images in greyscale.

Regarding the application of greater restrictions on the most common activities and the ones most easily detectable, we are not aware of any related work. Therefore, during the study of the state of the art, we have not found any precedent whose focus is exactly the same as this proposal: compare the effect of different improvement strategies on activity recognition systems. In consequence, a comparative analysis can be beneficial for future or existing works whose recognition is based on *skeletal tracking joint oriented devices*.

## 3. Difficulties of the Activity Analysis Based on Relevant Joints

In the evaluations previously done to the *E-BA-A* [5] we identified different types of activities that, due to their nature, are more difficult to monitorize. With the skeletal tracking joint oriented devices, depending of what kind of joint selection is made, the activity is more or less susceptible to monitoring errors. From a general perspective we can identify two traits to classify activities:

- *Global/Bounded Use of the Body*: The activity is characterized by using all or most of the monitored joints (*Global*) or by using a reduced subset of the same (*Bounded*).
- *Symmetry*: There are activities that use both halves of the body, either symmetrically or with an inverted symmetry, while others are characterized by using only one half of the body.

According to the monitored activities and the results of the evaluation, the movements that use the joints in a *global* way (like *Walk* or *Forward Bend*) obtained very good accuracy percentages. But in the case of activities that only took into account one subset (*bounded*), like for example the joints of the right arm, the results were significantly worse. This is mainly due to two factors: the number of relevant body parts and the postural coincidences.

The first factor is the influence that considering a higher or lower number of joints as relevant has on the *E-BA-A*. If an activity contemplates a greater number of joints, the effect that each joint can have individually, over the total result, is lesser. Therefore, the activities with less relevant joints are more exposed to errors. We will analyze this factor in depth during the development of Section 4, dedicated to the first of the considered strategies.

The second factor is the *postural coincidences* between different activities. There are different activities in which the position of certain joints and bones of the body is the same. When these similarities happen it makes it more difficult to distinguish the activities. This situation becomes specially critical when these *postural coincidences* occur between an activity in which the coincidental part of the body is relevant, and an activity in which it is not relevant.

These problems have motivated the search for strategies that would allow us to improve the results of the *E-BA-A*. During the next sections we will describe in detail the strategies that we have applied and analyzed. We will present how they have affected the results obtained by the *E-BA-A* in the evaluations that we have performed.

## 4. Strategy #1—Specialized Body Parts Analysis

### 4.1. Description of the Strategy

The possibility of *defining a personalized set of relevant joints for each activity*, gives the *E-BA-A* both positive and negative effects. By this reason, this feature should be used with precaution. We can summarize the effects in:

- *Positive Effects*
  ○ *Efficiency of the analysis*—Consider as relevant only a subset of the joints, reduces the computational load that the *E-BA-A* needs to analyze that activity. A smaller number of relevant bones leads to fewer mathematical calculations.
  ○ *Flexible use of the samples—*Using only the subset of relevant joints for the analysis, the *E-BA-A* can monitor the activity with a single sample of it, independently of the position of the rest of the joints. But if we consider the irrelevant joints too, it is possible that we will need several samples of the same activity depending on the positioning possibilities of the irrelevant joints.
- *Negative Effects*
  ○ *Each joint has greater influence—*If there are less *relevant bones*, each bone similarity has a greater impact in the final result. This fact could be positive or negative depending on the similarity value obtained. With less relevant bones, high bone similarity values benefits more the determination of high *body similarities*, but low bone similarities penalize much more the final result. This implies that any activity that has less relevant bones will be more exposed to possible precision errors that the sensor device can make.
  ○ *Avoid stable parts of the body—*There are certain parts of the body whose tracking is easier because its movement possibilities are limited (mainly the *trunk*). An easier tracking means an extra of *stability* (less vulnerable to precision errors) in the results. An activity with a reduced subset of relevant joints that do not consider those stable body parts, will be at a certain disadvantage.

Taking into account the negative effects, we could consider eliminating this feature (the possibility of *defining a personalized set of relevant joints for each activity*) as a possible solution to the problem (Section 3). But the inconvenience of this option is that we lose the interesting advantages that the current implementation of the *E-BA-A* offers, without discerning what effects (positive ones or negative ones) have more impact on the system. For this reason, we chose an intermediate approach for the first strategy applied.

The first strategy proposed, to improve the detection of the activities with a bounded use of the body, consists in: *studying the influence that body parts have on the recognition results*. The goal behind this intermediate approach is trying to reduce the negative effects that this characteristic of the algorithm introduces, but without rejecting the positive ones. If we can identify parts of the body that maximizes the obtention of high similarities, we can assign the *joints* of these parts as *relevant* (for the *bounded* activities).

To perform this study, we have defined several groups of different joints. As a matter of fact, one of the groups that has been contemplated in the evaluation is the complete set of supported joints (vC). We are attempting to reach a less radical solution than to use all (supported) joints of the body for all the activities. But we have chosen to include this scenario as well in order to study if its results (in comparison with the other groups of joints) clearly support its application.

In the next subsection we will explain what each group of joints consists of and what its goal is, as well as the evaluation dynamic of this improvement strategy.

### 4.2. Description of the Strategy Evaluation

The goal after this test is to find out if, on the activity recognition systems oriented towards *monitorization of joints*, it is more convenient to consider all or only those joints important for each activity. To study if it is more beneficial to include or omit the irrelevant joints, we will compare the recognition results obtained taking into account three different sets of joints.

The first set (vB) only considers the *joints of the extremities* used in the movement *without including the hands* (when the movement is done with the arm/s) *and the feet* (when the movement is done with the foot/feet). We omit these elements because in previous work, working with Kinect too, we observed that they tend to worsen the results. This way we want to check if just the elimination of these elements is enough to improve the recognition results of the movements whose relevant joints are a reduced subset. The joints corresponding to the shoulders and hips are also part of the set vB, as the union points between the trunk and the extremities belong to both.

The second set (vT) considers the same joints as the set vB but adding those belonging to the *trunk*. With this we try to test if considering the trunk (as it is one the parts with a more stable tracking) is viable to obtain high rates of accuracy without giving up the possibility of using samples of these movements from different positions. It is a compromise engagement, an option that involves more joints but is not as radical as to stop omitting irrelevant joints.

**Figure 1:**
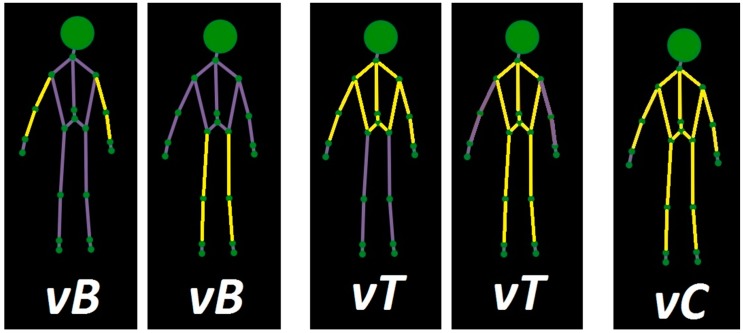
Visual representation of the sets vB, vT and vC.

The last set (vC) corresponds to *all the joints of the body* except hands, feet and head. Use of all the body theoretically eases the differentiation between activities. Therefore, with this set we try to test if accuracy rates in recognition improves remarkably in comparison with sets vB and vT. Figure 1 shows a visual representation of the three different sets, with the relevant bones in each case coloured in yellow.

As we have considered three different sets of joints, we have three different versions for each considered activity (each one of them with the relevant joints defined by each set). The four selected activities are: *Drink [D]* and *Grab Object [D]*, from the previous evaluation (and done with the arms), and *Leg Flexion [I]* and *Leg Circular Swing [I]* as new additions (that use the legs as the extremity). The activities identified with [D] are done with the right side of the body. These are the *asymmetric activities*, those that can be performed with either the left [I] or right side of the body, and therefore two versions must be recorded in the system, one for each half of the body.

One aspect that would be convenient to clarify is the *reason to select these activities*. Since the aim of this work is not to apply the activity recognition to a particular scenario, for a better understanding is important to remark that this selection is motivated for previous works about analyzing IADL activities in elderly people with and without dementia. One of the typical ways to evaluate cognitive impairment is through a *dual task*. The elder person performs one of those physical activities while doing some cognitive activity (for example, counting). But we selected these activities because we are looking for the classification improvement of those activities more difficult to detect through the skeletal tracking technology. Taking into account the results of previous work [5] these are the bounded activities (only a small subset of the joints is relevant). These four selected activities are good examples of activities difficult to classify for the *E-BA-A*. And we choose these four to have two of them performed with the arms and two of them performed with the legs. Besides, its relevant joints correspond only to the *extremities*. In consequence, they are ideal for the goals of this evaluation as they are compatible with the set vB.

The rest of the details of the evaluation can be seen in Appendix A, Appendix B, Appendix C and Appendix D, as these are aspects common to the rest of the evaluations performed in this work. Some of the aspects detailed in the appendixes are: the information about the bodies of the volunteers, the hardware used during the evaluations or the rest of the activities considered by the *E-BA-A* (apart from the selected ones for the evaluation).

### 4.3. Evaluation Results

**Figure 2:**
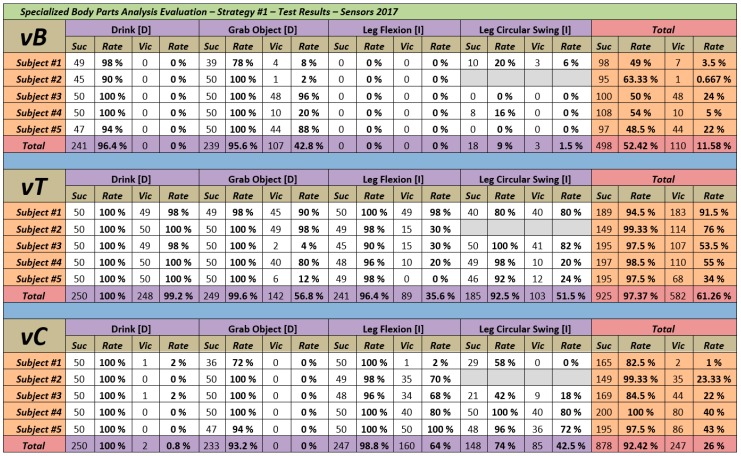
Specialized Body Parts Evaluation results for the sets vB-vT-vC.

Figure 2 shows the results obtained in this first evaluation. We can distinguish different types of information including the results from each volunteer for each of the movements, in addition to the totals by movement and totals per volunteer.

Each unit of information contains four fields. In first place, Suc indicates the number of times that the algorithm predicted correctly the movement performed by the participant. In second place, Vic indicates the number of times that that set of joints (vB, vT or vC) has obtained a similarity with the activity greater than the rest of considered sets. Both fields are accompanied by its corresponding success rate (Rate). In this first evaluation each test subject accomplished all activities a total of 50 times.

### 4.4. Analysis of the Results

The conclusions of this first evaluation were clear. The best approach in order to obtain better recognition results was the use of the set vT, meaning including the *trunk* as a relevant part of the body in all the activities. The set vC obtained high accuracy rates too (92.42%) but not as good as the results of the set vT (97.37%). Finally, the accuracy rates obtained by the set vB were far from the results obtained by vC and vT. The set vB obtained a global success rate of 52.42% highlighting the high contrast between the results obtained in the recognition of the activities performed with the arms (96.4% for *Drink [D]* and 95.06% for *Grab Object [D]*) and the activities performed with the legs (0% and 9% respectively for *Leg Flexion [I]*and *Leg Circular Swing [I]*).

## 5. Strategy #2—Stricter Restrictions for the Most Easily Detectable Activities

### 5.1. Description of the Strategy

The fact that the algorithm fails, confusing the performed activity with another of the most easily detectable, was already planned that it may occur [5]. The most basic and common activities, such as *Stand*, will have many joints positions in common (*postural coincidences*) with others. These kind of activities is what we call *sink* postures or movements. We selected the term *sink* to identify these activities due to the effect they produce on the results. They absorb samples as their own when they should be classified as a different activity. In other words, the *sink* activities obtain high percentages of similarity when the user is actually performing another activity.

These *postural coincidences* (Section 3) are particularly determinant when they occur with *bounded* activities. But *postural coincidences* are not the only factor that make it easier to obtain better results with *global* activities instead of *bounded* activities. The factors are listed below:

- *Postural coincidences*
  ○ *Irrelevant joints for an activity could be relevant for another—*Even though the information of the irrelevant joints of an activity is ignored by the *E-BA-A* in its analysis, these joints will be relevant for other activities. Therefore, it is possible that the position of these joints (irrelevant in one case and relevant in other) will be the same in both cases. This situation usually happens, thus the detection and differentiation between activities is more difficult. This is especially critical with the *bounded* activities because they have less joints to differentiate itself from the rest of the activities.
  ○ *Example—*To illustrate this we can describe as an example the activity *Grab Object*. This is an activity bounded with a high significance to the joints of the arm. If this activity is performed standing up, it can be easily confused with the activity *Stand* as the position for the rest of the joints is almost the same.
- *Greater exposure to precision errors* (Section 4.1 “*Each joint has greater influence*”)
- *Limitations of the sensor device*
  ○ *Computer vision devices nature—*The skeletal tracking joint oriented devices, due to their nature, are not capable of performing the tracking of the user’s body with the same level of precision for all kinds of actions. This means that, depending on the performed activity by the user, the device will have more or less difficulties to capture all the variations that have happened in the position of the joints of their body.
  ○ *Microsoft Kinect specific case—*Kinect is a device that is comfortable analyzing human bodies that are in front of it and looking to it. This is reasonable, given that the original application with which Kinect was made was to be an input device for the console *Microsoft XBOX 360*. A peripheral that allows the user to become a peripheral themselves. In this context, the interactions made by the users are always explicit because the user must stand in front of Kinect in order to be able to receive the feedback of videogames. But even in this situation Kinect has some limitations: *positioning joints imprecisions* even when the user is still, greater difficulties to *capture sudden variations* of position and the *inference of joints* when they are covered by another body part (like any computer vision device) and Kinect does not “see” them. This limitation has made it so that even some videogames developed by important companies in the sector, exclusive for Kinect, do not have a great reputation. The reason is that those videogames have not achieved a good user experience, because they fail to map the user’s action a significant number of times.

Once we have exposed the three factors that hinder the recognition of *bounded* activities, it is easy to understand the three opposite factors that characterize *sink* activities. These factors are: (1) they use the body in a *global* way; (2) its monitoring is not demanding for the sensor device; (3) if there are *postural coincidences*, these are resolved in their favor, as there is a great chance that those joints will be relevant for the *sink* activity. Of the total number of activities considered (Appendix A) we consider as sink: *Stand*, *Stay Seated*, *Walk* and *Walk Backwards*. All of them have in common that they are activities performed smoothly, with a big set of relevant joints and without body parts trajectories that cover others.

Taking all of this into account, we have a group of activities (*sink*) with a greater probability that its elements will be selected as a prediction, and another group (*bounded* activities) with difficulties for being detected. In consequence, the second strategy proposed to improve the detection of the last ones, consists in *applying some sort of restriction to the sink activities*. The goal of this strategy is to reduce the number of occasions in which a *sink* activity is chosen as a prediction by the *E-BA-A* when it is really not being performed. The idea for it is to introduce a barrier. If the barrier value is not surpassed, the system will consider that the *sink* activity is not similar enough to be considered the prediction for that instant (even if it has obtained the higher similarity percentage).

In this respect we have introduced the concept of *minimum limit of prediction* in the past [5]. Its original objective was to discern those situations in which the user was doing an *unknown activity* for the system. So, why not apply this idea on another level? Instead of defining a single limit that has to be surpassed by any activity to be considered for prediction, establish a stricter and more exclusive limit for sink activities. But, which *value* do we use? *One* or *more* limits? To answer these questions, we have elaborated a preliminary evaluation.

### 5.2. Description of the Pre-Evaluation

To obtain the necessary data to select the limit to apply, the pre-evaluation is divided in two parts. The first part consists of performing the four sink activities considered (*Stand*, *Stay Seated*, *Walk [D]* and *Walk Backwards [D]*) several times in order to analyze which similarity percentages are obtained approximately when the user is really doing a sink activity. The second part consists of performing the four activities whose recognition rates we are trying to improve (*Drink [D]*, *Grab Object [D]*, *Leg Flexion [I]* and *Leg Circular Swing [I]*) but analyzing which similarity is obtained in each case with each one of the sink activities. This way we can make an approximation of what similarity percentages are obtained by the four sink activities when they are not being performed.

Through the results of both parts of the pre-evaluation we attempt to discover the interval of values in which we can choose the limit or limits to apply. The rest of the details of the pre-evaluation coincide with the rest of the performed tests and can be seen in Appendix A, Appendix B, Appendix C and Appendix D.

### 5.3. Pre-Evaluation Results

**Figure 3:**
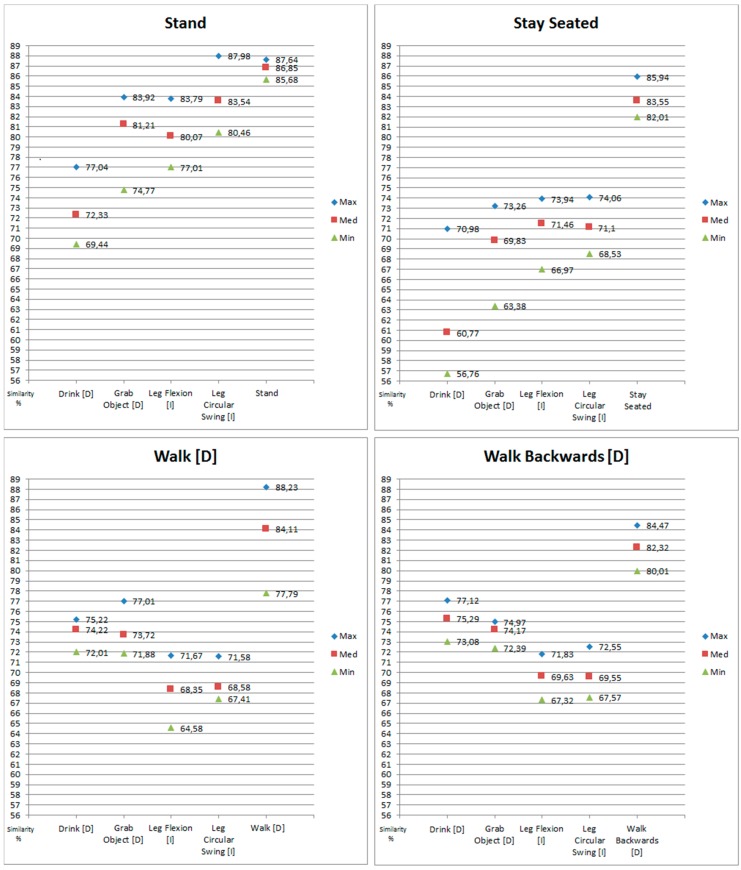
Graphical Representation of the Limit Selection Pre-Evaluation Results.

Through this pre-evaluation, what we are looking for is to delimit the intervals of values in which we can choose a limit that could correctly perform our goals. With the performance of the sink activities (second part of the test) we search the upper limit of the interval. The greatest values of similarity that can be reached doing the sink activities. Meanwhile, the similarity values obtained by the sink activities, when the performed activity was not one of them (first part of the test), indicates us the lower limit of the interval. The similarity values that should not surpass the limit if we want to obtain the desired behavior. To ease the comprehension of the limit selection process, Figure 3 shows the results of the pre-evaluation visually.

In our search of the *minimum limit or limits of prediction* we have considered the maximum, minimum and mean of the values that defined the selection interval. The reason for this is that, depending on the size of the interval (the difference between the values that suggest the upper limit and those that suggest the lower limit), we will have to use one of the different statistical values as the definite interval delimiters.

The ideal situation would be that there would be a big interval of values (the wider the better) between the minimum of the upper limit candidates and the maximum of the lower limit candidates. If this happens, we will have more guarantees about the correct functioning of the limit. Meaning, the limit would be surpassed when it has to be surpassed (the user is really performing a *sink* activity), and it wouldn’t be surpassed when it hasn’t been surpassed (the user is not performing a *sink* activity).

**Figure 4:**
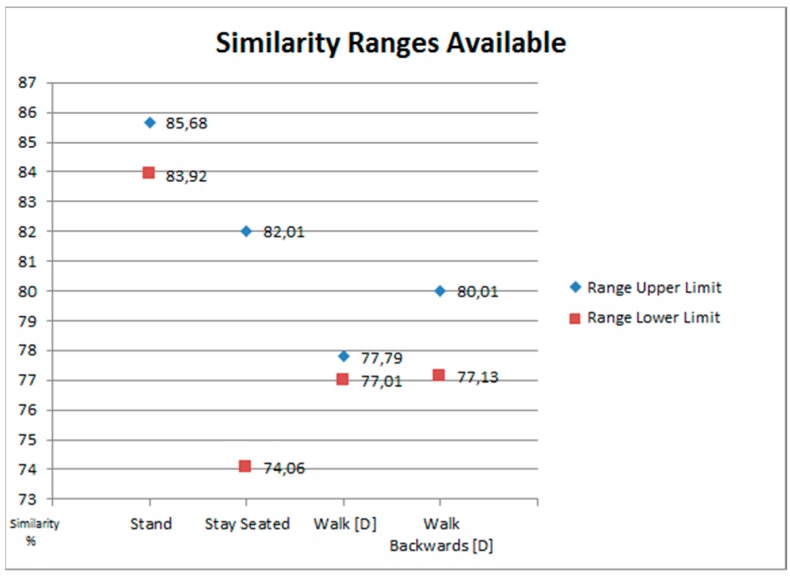
Available ranges of values for selecting *minimum limits of prediction*.

An example of this ideal situation has happened with the results of the sink activity *Stay Seated*. As it can be seen in Figure 3, and with more detail in Figure 4, there is a big range of available values for the activity *Stay Seated*. A big range will facilitate that the selected limit differentiates the cases in which the sink activities are being done, and when they are not. This situation also happens with the activities *Walk [D]* and *Walk Backwards [D]* but with the difference that the available values interval is much smaller.

In the case of *Stand* the situation is more complicated. There is not an interval of values between the minimum of the upper limit candidates and the maximum of the lower limit candidates. This is because the maximum obtained similarity, when the sink activity is not being made, is greater than the minimum obtained similarity when the sink activity was actually being performed. When this happens it is necessary to adjust the selection criteria for the values that will define the selection interval. The consequence is that, even assigning a *minimum limit of prediction*, it will be much more difficult to discern when this activity is really being done and when it is not.

Figure 4 shows the selected upper and lower limits. The *minimum limit of prediction* will be a value that is within the interval defined by those limits. But given the results, it would not be advisable to choose to use a single limit for all four sink activities. In the case of *Stay Seated*, *Walk* and *Walk Backwards* they share a part of the interval’s values. So, it would be feasible to choose a single limit to control all three activities. But in the case of *Stand* its interval is very different from the rest. In consequence, the chosen limit for the other three would not work.

**Figure 5:**
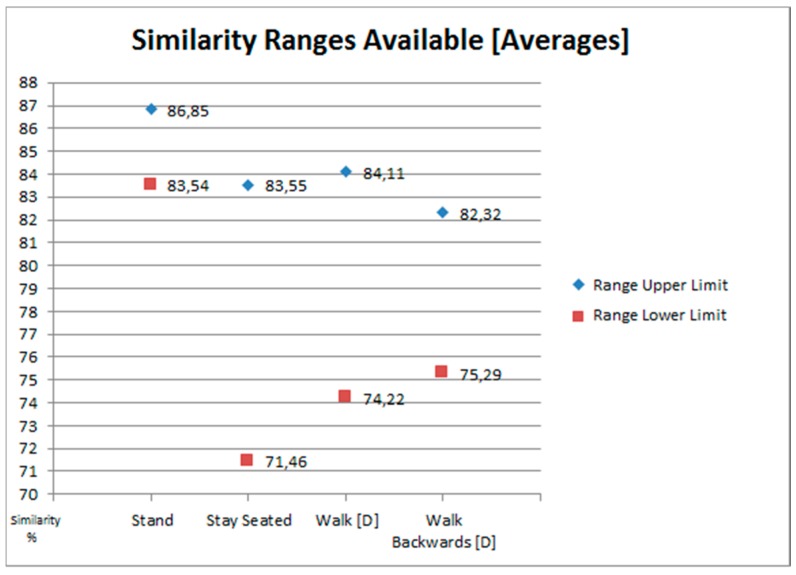
Consequences of widening the range of values of *Stand*.

Even if we adjust the selection of the interval for the case of *Stand* using mean values (instead of maximum and minimum to choose the limits), the interval increases but not enough (Figure 5).

Taking the situation into account, we decided to use a personalized limit for each sink activity. This way, each limit could work better individually as it is designed only to control a concrete activity. Moreover, with a large number of sink activities, we will probably have the same problem (there are not shared intervals for all cases). Table 1 has the selected values as *minimum limit of prediction* for each one of the *sink* activities analyzed in this pre-evaluation.

### 5.4. Description of the Strategy Evaluation

Once we have described the preliminary evaluation, the main evaluation of this second strategy is described as follows. With this evaluation we have two objectives:

(1) *Test if the selected limits are too strict*: Test if even applying a restriction of having to surpass a limit, the sink activities are still being detected when they are performed.
(2) *Test if the selected limits fulfill their purpose*: Test if the limits prevent that sink activities are selected as a prediction when they are not being performed.

To achieve this, every participant has done a total of 25 instances of both the sink activities and the activities whose recognition we are trying to improve. For this evaluation, we have begun from the base of the results previously obtained by the set vB (Section 4.4). As its results have demonstrated that eliminating hands and feet as relevant joints is beneficial, the samples used in this evaluation do not include these body parts. The rest of the details of the evaluation, common to the rest of the performed tests, can be seen in detail in Appendix A, Appendix B, Appendix C and Appendix D.

### 5.5. Evaluation Results

**Figure 6:**
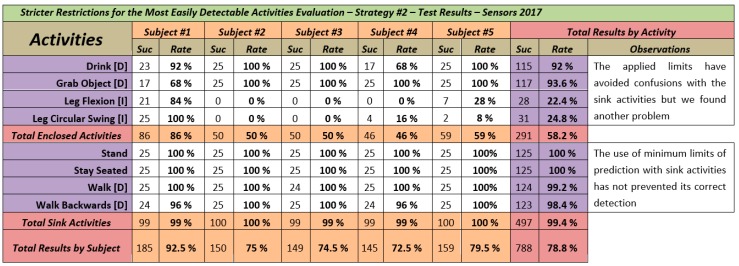
Stricter Restrictions for the Most Easily Detectable Activities Evaluation Results.

Figure 6 shows the results obtained from each participant for each of the activities in addition to the totals by activity and totals per volunteer. Each unit of information (intersection between subject and activity) contains two data. Suc indicates the number of times that the algorithm predicted correctly the movement performed by the voluntary. This field is accompanied by his corresponding success rate (Rate) taking into account that the volunteers performed all activities a total of 25 times (a total of 1000 activities performed during the evaluation).

Unlike the other strategies, in this second one, we do not use the term Vic. The reason for that is we cannot use Vic in this kind of strategy. Here we have only applied limits to the sink activities. In this strategy, we do not have different sets to compare. As Vic indicates when one set obtains higher similarity than others, it does not make sense.

### 5.6. Analysis of the Results and Comparative with the Results of the Previous Strategy

We will analyze the effect that the *minimum limit of prediction* have had over the *sink* activities. As it can be seen in Figure 6, the application of these restriction on these types of activities that are easier to detect, has had no negative impact on the results. As a matter of fact, the four activities have had accuracy rates greater than 98%. Even in the case of *Stand* and *Stay Seated* the rate has reached 100%. Analyzing the results from the point of view of the participants, the results have been equally positive, with all participants obtaining an accuracy rate between 99% and 100% while doing the sink activities.

Regarding the *bounded* activities, there has been improvement in the accuracy rates, but not as much as expected. The activities whose relevant joints are only in the arms (*Drink [D]* and *Grab Object [D]*) have gotten very good results (92% y 93.6% of accuracy rate respectively). If we compare these numbers to the ones obtained by these activities through the set vB (96.4% and 95.6% respectively), the results are slightly worse, but the differences are not so great as to be significant. Especially considering that this evaluation has been more aggressive than the evaluation of the first strategy. Performing less instances of the activities, the detection errors penalize the final accuracy rate so much more.

The activities performed with the legs (*Leg Flexion [I]* and *Leg Circular Swing [I]*) have obtained very low accuracy rates (22.4% and 24.8% respectively) but the result is significantly better than the one through set vB (0% and 9% respectively) though that is not improvement enough. It is worth mention the results obtained by the participant #1. An accuracy rate of 84% in *Leg Flexion* and 100% in *Leg Circular Swing*.

Given the results, one would think that the application of limits has not worked as it should have, but this has not been the case. In order to control the development of the evaluation and collect the results, we developed an application. This application allows us to observe the evolution of the experiment, the results so far and with which activity it is being confused in case of a failure. All this at *interactive time* (also known as *operational time*) while the participants performed the activities. When we started to see that there were many mistakes, we started to pay attention to the predicted activity in the failure cases. We wanted to know if the confusion was due to any of the sink activities in particular, and if maybe there should be a change in the value of the limit selected.

But it was not like that. Most of the confusions in the detection of *Leg Flexion* and *Leg Circular Swing* happened with *Grab Object*, bumping down both activities to the second place of most similarity obtained (in most cases by a negligible difference in percentage, less than 0.2%). Therefore, the limits were working correctly, avoiding that sink activities would be the prediction even when obtaining a higher similarity percentage. But we did not think this could happen.

Seeing the results of *Grab Object* in the evaluations of the first and second strategy, it is obvious that it is an activity that is easier to detect than those performed with the legs (once the strategies proposed in this paper have been applied). But it is not an activity that we currently consider a sink activity. It is true that it fits the criteria of not being done suddenly. But on the other hand, its number of relevant joints is quite small, and it is an activity very prone to having postural coincidences that negatively affect its results.

Based on what happened, we can conclude that the strategy of applying limits to sink activities is an interesting idea. An idea that can contribute to improve recognition, but it needs to be refined.

The explanation for what may have happened is as follows. When asking the participants to perform an activity, they are shown how to make it, but avoid conditioning its performance. The only stricter guideline is that they must follow the speed of the activity to perform. There can be differences regarding the master sample, but the speed cannot differ significantly (the management of these differences is part of the future work). The theory is that, when performing activities with the legs, the movements done by the arms of the participants unconsciously to maintain balance, may have been confused with the movement *Grab Object*. This attached to the oscillations in which Kinect frequently incurs in, may have been the reason.

Lastly, if we compare on a general level the performance of both strategies (*specialized body parts analysis* vs. *stricter restrictions for the most easily detectable activities*) undoubtedly the first of those is the winner. Thanks to the excellent results obtained through the set vC as well as the set vT. The application of the *minimum limits of prediction* (personalized to each sink activity) has demonstrated to be an interesting strategy. One that works in the sense of limiting the prediction of sink activities only when they are being done (without reducing its accuracy rate). But its application by itself cannot be considered as a solution to the problem we are trying to solve.

Given the virtues that applying the limits has demonstrated, it is a strategy that maybe, with a little bit more refinement (and with a detailed study of the exact reasons of what has happened with *Grab Object*), it can end up as a useful tool for activity recognition.

## 6. Strategy #3—Combination of Body Parts and Stricter Limits

### 6.1. Description of the Strategy

Once we applied the two previously proposed strategies, the next logical step would consist in combining both strategies. The *specialized body parts analysis*, through the set of joints vT, has proven to be a possible solution to the problem of detect *bounded* activities (Section 3). On the other hand, the use of *stricter restrictions* for the most easily detectable activities has not worked as we expected, (recognition rates doesn’t improve enough) but it has positive aspects (the limits work properly). For this third strategy, we have combined both approaches, with the aim of strengthening the recognition algorithm (*E-BA-A*) to have the advantages of both.

### 6.2. Description of the Strategy Evaluation

The dynamic of the evaluation is the same as in the first strategy (Section 4.2). The difference is that, apart from the sets vB, vT and vC, the *minimum limits of prediction* assigned to the sink activities are also present, and can be seen in Table 1.

### 6.3. Evaluation Results

**Figure 7:**
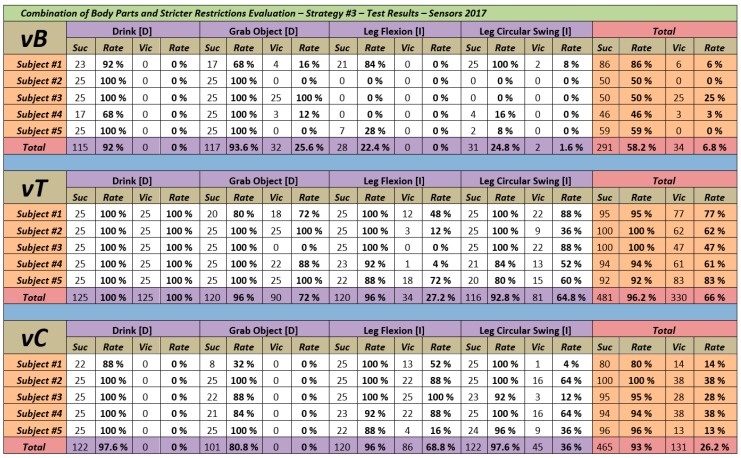
Combination of Body Parts and Stricter Limits Evaluation Results.

The interpretation of Figure 7 and its results is equivalent to the interpretation of the results from the first evaluation (Section 4.3). Suc indicates the number of times that the algorithm predicted correctly the movement performed by the participant. Vic indicates the number of times that that set of joints (vB, vT or vC) has obtained a similarity with the activity greater than the rest of considered sets. The only difference is that, in this occasion, each volunteer has performed each activity a total of 25 times instead of 50. This way the evaluation is more aggressive, as any mistake will penalize the final accuracy rate much more than if we have a larger number of instances.

### 6.4. Analysis of the Results and Comparative with the Results of the Previous Strategies

The obtained results are, in a way, aligned with those obtained during the *specialized body parts analysis evaluation* (Section 4.4). As it happened then, the set vB is able to obtain high rates of success but only in the recognition of the activities performed with the arms (92% for *Drink [D]* and 93.6% for *Grab Object [D]*). In the case of activities whose relevant joints are concentrated on the legs, thanks to the combined use of the *minimum limits of prediction*, the results have experimented an improvement (*Leg Flexion [I]* has gone from 0% to 22.4% and *Leg Circular Swing [I]* from 9% to 24.8%). But this improvement is not enough. The global accuracy percentage has also improved, going from 52.42% for the set vB to 58.2%. Hence, the option of combining the set vB with the use of the *minimum limits of prediction*, allows to improve the results of the set vB on its own. But it is not a viable option to obtain high accuracy rates (more details on why the limits have not improved more the results of the set vB in Section 5.6).

With the sets vC and vT the situation is also the same of what previously happened. The use of the set vC, in combination with the *minimum limits of prediction*, is recommended as it has obtained an overall success rate of 93%. The limits have contributed to improving its results, as in the original evaluation, the global accuracy rate was of 92.42%. But again, and as it happened in that evaluation, the drawback is that even though it is a very good rate, it is not the set that has achieved the best results. The best global success rate obtained during the evaluation has been 96.2%, corresponding to the set vT.

In this last case, the accuracy rate has been somewhat lower to the original (97.37%). But given that the evaluation has been more aggressive (as we had a smaller number of instances performed) this does not imply that the results from the set vT worsen when used in combination with the limits. Given the obtained results, we can conclude that the use of limits as a support tool contributes to the improvement of activity recognition.

## 7. Discussion

One of the limitations of this work is the size of the test population. In order to perform the evaluations, we had the collaboration of five participants. It is true that the number of participants is small, but on the other hand, the number of samples classified by the *E-BA-A* during the evaluations was huge. The five participants performed a total of more than 3500 samples of the considered activities in the three evaluations.

Besides, in a system with the characteristics of the *E-BA-A*, it is much more important to have many samples to classify, rather than many different participants; because these ones are not part of any training set. With a single sample of each activity, the system is classifying, with good results, thousands of samples performed by five different persons. This makes it not so critical to have a large test population. Furthermore, even though having only five participants, characteristic and notable differences have been observed between the different strategies applied.

Regarding the extrapolation of the results to other proposals, the results and clues obtained in this work could be extrapolated mainly to other systems based on the same kind of technology. This is due to the particularities of the skeletal tracking joint oriented devices. But there are some specific lessons learned that can be theoretically generalized. During the application of the first strategy, the results clearly indicate that the use of the *trunk* can favor a better recognition of the activities. These findings about the significance of each body part in the recognition can be useful for systems not necessarily based on skeletal tracking joint oriented devices.

## 8. General Conclusions and Future Work

Starting with the first strategy, its analysis results (Section 4.4) clearly indicate that the best option to analyze *bounded* activities is used, alongside the joints of the extremities, also the joints of the trunk. The trunk eases the obtention of high similarity percentages because its monitorization is more precise. It is a part of the body whose range of movements is more limited, facilitating its tracking with joint oriented devices.

The trunk provides stability to the results. It also reduces the factor that, to contemplate less *relevant bones*, they have more influence on the obtained similarities. And at the same time, they are more exposed to the sensor device possible failures. By including the trunk, we achieved that the activity has an *intermediate amount of relevant joints* (and therefore *relevant bones*), which ensures a balanced weight distribution in the obtaining similarities process.

But the use of the trunk alongside the extremities has not only been beneficial regarding the obtention of better rates of success. It is also useful to improve the victory percentage. Getting the set vT to obtain both a *greater overall success rate* (97.37%) as well as the *highest percentage of wins* (61.26%). As it is able to obtain higher similarities (than the rest of alternatives sets), potentially it will also obtain greater differences regarding the similarities obtained for the rest of the activities that have not been done by the user. As a result, obtaining greater differences with the rest of activities, theoretically eases the differentiation between activities.

Regarding the application of greater restrictions for the most easily detectable activities (second strategy), it has not worked as we expected. It is an interesting idea because the restrictions that we applied worked well. The limits were able to avoid the prediction of *sink* activities when they were not being performed. But, by itself, it is not a solution to improve the recognition of *bounded* activities.

But on the other hand, the results of the third strategy, indicates us that the use of the limits could be useful as a support tool. The addition of the limits, in combination with the first strategy, improves the recognition rates of the sets vB and vC and the success percentages of the set vT (this last one going from 61.26% to 66%).

The results obtained in this work pretend to be some first indications about what considered strategy is better. Taking into account that the population of volunteers that we have used is small, we offer some preliminary clues for other researchers who are working on systems based on the same kind of technology.

Apart from increasing the size of the test population, future work goes through including distances into the *E-BA-A*. This way we will be able to improve the results obtained by this last one enriching the information it works with. Going from only using the angles to detect the activities, to combining the angles with the distances the joints have moved. This measure could be the solution to the conflict experienced with the activity *Grab Object* (Section 5.6) as to reach a decision, the distance moved by the leg joints would also be taken into account.

Other lines of future work consist in maximizing the use of the algorithm collaborating with other authors. The idea is to add value to other works. On the one hand we will combine this system with an affective avatar [28] in order to give this last one information about the activities that the user is doing. On the other hand, we will develop a videogame whose main mechanic will be the realization of physical activities. This will allow to analyze the relationship between the game mechanic and the executive function [29]. The activities performed during the game will be analyzed through the *E-BA-A*.

## Figures and Tables

**Table 1:** Minimum Limits of Prediction assigned to the Sink Activities.

Activity	Minimum Limit of Prediction
Stand	**85.4%**
Stay Seated	**78.0%**
Walk [D]	**78.0%**
Walk Backwards [D]	**79.0%**

## Appendix A. Activities Considered during the Performance of the Evaluations

Each of the evaluations recorded in this paper has as the object of study a group of activities that is not the same in all of the cases. Also, none of the cases in that group of activities coincides completely with the contemplated activities in the system’s data base. Therefore, even though we evaluate the development of each activity that is of interest in each evaluation, this does not mean that they are the only activities that are considered by the *E-BA-A*. The goal is to test if the proposed solutions allows to improve the recognition of activities with a more reduced subset of relevant joints. Hence, it is necessary that the algorithm contemplates more activities in order to make the task more complicated. The total of activities considered by the *E-BA-A* in all of the performed tests can be seen in Table A1 (which contemplates the characteristics previously explained about *Symmetry* and *Global/Bounded Use of the Body*).

**Table A1:** Taxonomy of the considered activities by the ***E-BA-A*** during the tests.

	Symmetric	Asymmetric
Global	Forward Bend Sit Down Stand Up Stand Stay Seated	Walk Walk Backwards
Bounded	Squat	Drink Grab Object Leg Flexion Leg Circular Swing

It is worth mentioning the case of the activities *Walk* and *Walk Backwards*. As it can be seen in the evaluation corresponding to the second strategy considered for this work (Section 5), these activities are signaled with a *[D]*. The reason is that the samples were recorded starting the walk with the right leg. This detail is indifferent as long as the user that is being monitored walks more than one stride cycle. But if only one is made, the leg with which the user starts to walk with has to be taken into account. Because of this, in the future, we will also include in the system’s data base a sample of the activities *Walk [I]* and *Walk Backwards [I]*.

## Appendix B. Body Information of the Participants of the Evaluations

The evaluations have been done thanks to the participation of five volunteers of different ages, genders (two men and three women) and complexions. These last are detailed in Table A2. The participants have no disabilities nor specialized background in any athletic discipline.

**Table A2:** Test Subjects Corporal Information.

Test Subject	Height	Shoulder Width (cm)	Hip Width (cm)	Age
#1	1.60	39	45	26
#2	1.81	50	42	57
#3	1.56	44	38	56
#4	1.68	37	34	23
#5	1.84	45	44	26

The samples used by the *E-BA-A* (only one for each considered activity) for training were performed only by the test subject #5. This means that we tested the performances of the subjects #1, #2, #3 and #4 with the information of only one person (the test subject #5).

## Appendix C. Hardware Used During the Evaluations

The hardware used during the evaluation was a standard desktop computer whose main components correspond with a RAM of 8 GB DDR4 and an Intel Quad-Core i5-6500HQ processor operating at 2.3 GHz. The *Extended Body-Angles Algorithm* not requires specialized hardware and even it is capable of running on older hardware configurations, as it is explained in greater detail in previous publications [5]. Depending on the performance of the used equipment, the main difference is the sampling rate with which the sensor device is capable of perceive its surroundings. And in consequence, the samples that the algorithm processes per second is different too.

## Appendix D. Particularities of the Evaluations and Data Gathering Protocol

In this work, we have used an original method, named *E-BA-A*, in order to do the classification of the activities, whose successful performance was validated in previous works [5]. At first sight, this method could be identified as just another proposal based on machine learning techniques. However, and considering the Davis and Marcus taxonomy [6], this kind of interaction cannot be considered machine learning. Actually, we are proposing a commonsense reasoning method based on mathematical analogies for activity recognition. For this reason, it is convenient to clarify some aspects which sometimes can be confusing. These two aspects, related with the development of the evaluations, are: the fact that *there is no need to record* the performed activities and the *simultaneous obtention* of the results.

In more detail, for a better understanding of this recognition algorithm, it is important to remark that the *E-BA-A* do not need to record large data corpora to obtain the representation of the activities. In consequence, we do not create a dataset with multiple samples of each activity in order to process that information later. The *E-BA-A* is capable of classifying activities using only one sample of each one through mathematical analogies. This means that, when the participants performed the activities, all the instances are directly classified by the *E-BA-A*. The thousands of instances of the considered activities performed by the participants (more than 3500 taking into account all the evaluations) are only for test purposes, not for training.

On the other hand, and taking the previous point into account, we obtain the results of the evaluations in a simultaneous way, during its realization. The *E-BA-A* works at interactive time (also known as operational time). This means that, the results of the evaluation and the predictions of the *E-BA-A*, are obtained at the same moment that the participant performs the activity.

Once we explained these particularities, we are going to detail the *data gathering protocol*. As the *E-BA-A* is not a system based on machine learning, the concepts of *training set* and *test set* are a little bit blurred. The reason for that, as we previously introduced in the paper, is that the *E-BA-A* only needs one sample of each considered activity (training set). In consequence, all the activities performed during the evaluations are for test purposes only (test set). But we are going to use these concepts to facilitate the comprehension of the data gathering. In order to build the training set (only one sample of each activity), before the beginning of the evaluation, we asked one of the participants to perform every considered activity only one time. We recorded this performance in the database of the *E-BA-A* using *Microsoft Kinect* (specifically its skeleton stream). Each element of the database contains the position of the joints of the body in each frame of the activity. This way, the *E-BA-A* will use these unique samples of each activity to do the classification of future samples.

Regarding the building of the test set, during the evaluations, we asked the participants to perform the repetitions of the activities in front of Kinect. But these repetitions were not recorded. This means that, all of these repetitions are used as a test set, but are used on the fly. Thanks to the capabilities of the *E-BA-A*, in the exact moment in which the participant finishes the performing of every repetition, we obtained the classification result of that repetition.
